# The development of the Physical Activity Environment Policy Index (PA-EPI): a tool for monitoring and benchmarking government policies and actions to improve physical activity: Catherine Woods

**DOI:** 10.1093/eurpub/ckac129.270

**Published:** 2022-10-25

**Authors:** C Woods, L Kelly, K Volf, P Gelius, S Messing, S Forberger, J Lakerveld, N den Braver, J Zukowska, PEN consortium

**Affiliations:** 1 Physical Activity for Health Research Cluster, University of Limerick, Limerick, Ireland; 2 Department of Sport Science and Sport, Friedrich-Alexander-Universität Erlangen- Nürnberg, Erlangen, Germany; 3 Leibniz Institute for Prevention Research and Epidemiology, Bremen, Germany; 4 Department of Epidemiology and Data Science, Amsterdam UMC, VU University Amsterdam, Amsterdam, Netherlands; 5 Upstream Team, VU University Amsterdam, Amsterdam, Netherlands; 6 Faculty of Civil and Environmental Engineering, Gdansk University of Technology, Gdansk, Poland

## Abstract

**Background:**

Insufficient physical activity (PA) is a global issue for health. A multifaceted response, including government action, is essential to improve population levels of PA. The purpose of this study was to develop the ‘Physical Activity Environment Policy Index’ (PA-EPI) monitoring framework to assess government policies and actions for creating a healthy PA environment.

**Methods:**

An iterative process was undertaken. This involved a review of policy documents from authoritative organisations, a policy audit of four European countries, and systematic reviews of scientific literature. This was followed by an online consultation with academic experts (N = 101; 20 countries, 72% response rate), and policymakers (N = 40, 4 EU countries). During this process, consensus workshops where quantitative and qualitative data alongside theoretical and pragmatic considerations were used to inform PA-EPI development.

**Results:**

The PA-EPI is conceptualised as a two-component ‘policy’ and ‘infrastructure support’ framework. The two components comprise eight policy and seven infrastructure support domains. The policy domains are education, transport, urban design, healthcare, public education (including mass media), sport-for-all, workplaces and community. The infrastructure support domains are leadership, governance, monitoring and intelligence, funding and resources, platforms for interaction, workforce development, and health-in-all-policies. Forty-five ‘good practice statements’ (GPS) or indicators of ideal good practice within each domain concludes the PA-EPI. A potential eight-step process for conducting the PA-EPI is described.

**Conclusions:**

Once pre-tested and piloted in several countries of various sizes and income levels, the PA-EPI GPS will evolve into benchmarks established by governments at the forefront of creating and implementing policies to address inactivity.

